# The Association of COVID-19 and Mortality in Hospitalizations With Coronary Artery Bypass Graft

**DOI:** 10.1016/j.jscai.2025.103605

**Published:** 2025-05-13

**Authors:** Godfrey Tabowei, John Garza, Ayman Fath, Ahmed Bashir Sukhera, Oboseh John Ogedegbe, Gabriel Alugba, Samuel Dadzie, Ooreoluwa Fasola, Meron Tesfaye, Evbu Enakpene, Anand Prasad

**Affiliations:** a Department of Internal Medicine, Texas Tech University Health Sciences Center Permian Basin, Odessa, Texas; b Texas Tech University Health Sciences Center School of Medicine - Permian Basin, Odessa, Texas; c Department of Cardiology, University of Texas Houston, Houston, Texas; d Department of Internal Medicine, Trinity Health-Ann Arbor Hospital, Ann Arbor, Michigan; e Department of Internal Medicine, Englewood Hospital and Medical Center, Englewood, New Jersey; f Department of Internal Medicine, Piedmont Athens Regional Medical Center, Atlanta, Georgia; g Department of Internal Medicine, Texas Tech University Health Sciences Center, Odessa, Texas; h Texas Tech University Health Sciences Center School of Medicine, Odessa, Texas; i Department of Cardiology, Texas Tech University Health Sciences Center Permian Basin, Odessa, Texas

**Keywords:** coronary artery bypass graft, COVID-19, length of hospital stay, mortality

## Abstract

**Background:**

COVID-19 is associated with a higher burden of cardiovascular morbidity and mortality. The association of COVID-19 and mortality in hospitalizations with coronary artery bypass graft (CABG) has not been determined.

**Methods:**

We conducted a population-based cohort study of the association of COVID-19 and mortality in hospitalizations with CABG using the Texas Inpatient Public Use Data File over the period Q2 2020 through Q4 2023. The primary exposure was a diagnosis of COVID-19, and the primary outcome was in-hospital mortality. Short-term mortality and total length of stay were used as secondary outcomes. The primary analysis approach was overlapping propensity score weighting with treatment weighting and inverse probability of treatment weighting applied as alternative analyses. Results are reported as adjusted risk ratio (aRR) and 95% CI. The adjusted risk difference and 95% CI are provided as an alternative effect size measure.

**Results:**

A total of 47,501 hospitalizations with a procedure code for CABG were identified, of which 509 (1.1%) had COVID-19. CABG hospitalizations with vs without COVID-19 had higher comorbidity index (2.01 ± 1.65 vs 1.65 ± 1.61), more frequent need for invasive mechanical ventilation (12.8% vs 8.5%), had higher rates of myocardial infarction (65.0% vs 45.6%), higher rates of congestive heart failure (53.4% vs 40.1%), and higher rates of acute kidney injury (35.6% vs 24.3%); *P* < .0001 for each comparison. CABG hospitalizations with COVID-19 had higher unadjusted in-hospital mortality (6.3% vs 2.2%) and unadjusted length of stay (16.2 ± 11.6 days vs 9.6 ± 6.8 days), compared with those without COVID-19. In adjusted analysis, COVID-19 was associated with a 53.9% increase in the risk of in-hospital mortality (aRR, 1.5394; 95% CI, 1.0836 to 2.1870) and a 40.3% increase in length of stay aRR 1.4028 (95% CI, 1.3138 to 1.4978).

**Conclusions:**

COVID-19 was strongly associated with increased mortality in hospitalizations with CABG during the pandemic. The association weakened year over year and by 2023 was no longer present. COVID-19 has remained strongly associated with increased length of stay in hospitalizations with CABG including after the conclusion of the pandemic.

## Introduction

Coronary artery disease continues to be a leading cause of death in the United States despite advancements in the treatment of acute coronary syndrome and cardiovascular risk factors. Coronary artery bypass graft (CABG) remains the most common type of open-heart surgery, with about 400,000 surgeries being performed yearly in the United States.^1^ For patients with severe coronary artery disease, CABG is a life-saving technique that has accounted for approximately 7% of the overall drop in deaths from coronary heart disease.^2^ Since the outbreak in 2020, SARS-CoV-2 and associated COVID-19 have been reported to be responsible for 6,935,240 hospitalizations and 1,190,122 deaths in the United States.^3^ Although the virus primarily affects the respiratory system, it can also have impacts on other systems with cardiovascular complications affecting up to 30% of patients with COVID-19.^4^ The mechanism of SARS-CoV-2–induced cardiotoxicity is believed to be due to the effect of cytokine release driven by immune response as well as direct cytotoxic effects of the virus. This leads to a cascade of events including severe inflammation, plaque destabilization, and a prothrombotic state with a risk of cardiac dysfunction.^5^ Cardiovascular complications frequently observed with COVID-19 infection include cerebrovascular disorders, arrhythmias, ischemic and nonischemic heart disease, pericarditis, myocarditis, heart failure, and thromboembolic events.^6^ There are limited data on outcomes of CABG during the COVID-19 pandemic.^7^^,^^8^ We sought to evaluate the clinical outcomes of CABG for patients with COVID-19 compared with those undergoing CABG in the same period without COVID-19.

## Materials and methods

### Study design and data sources

A retrospective and population-level study was performed using the Texas Inpatient Public Use Data File (TIPUDF).^9^ The TIPUDF is a publicly available and deidentified data set, and the study was determined to be exempt from review by the Texas Tech Health Sciences Center Institutional Review Board. The TIPUDF is an administrative data set provided by the Texas Department of State Health Services that summarizes information from nonfederal hospitals including demographics, diagnoses, procedures, and hospital disposition, capturing an estimated 97% of hospital discharges in the state. Hospitalizations are used as the unit of analysis because the TIPUDF provides hospitalization-level and not patient-level information, preventing the identification of repeated admissions. We have followed the Strengthening the Reporting of Observational Studies in Epidemiology guidelines.^10^

### Patients and variables

#### Cohort derivation

**Figure 1:**
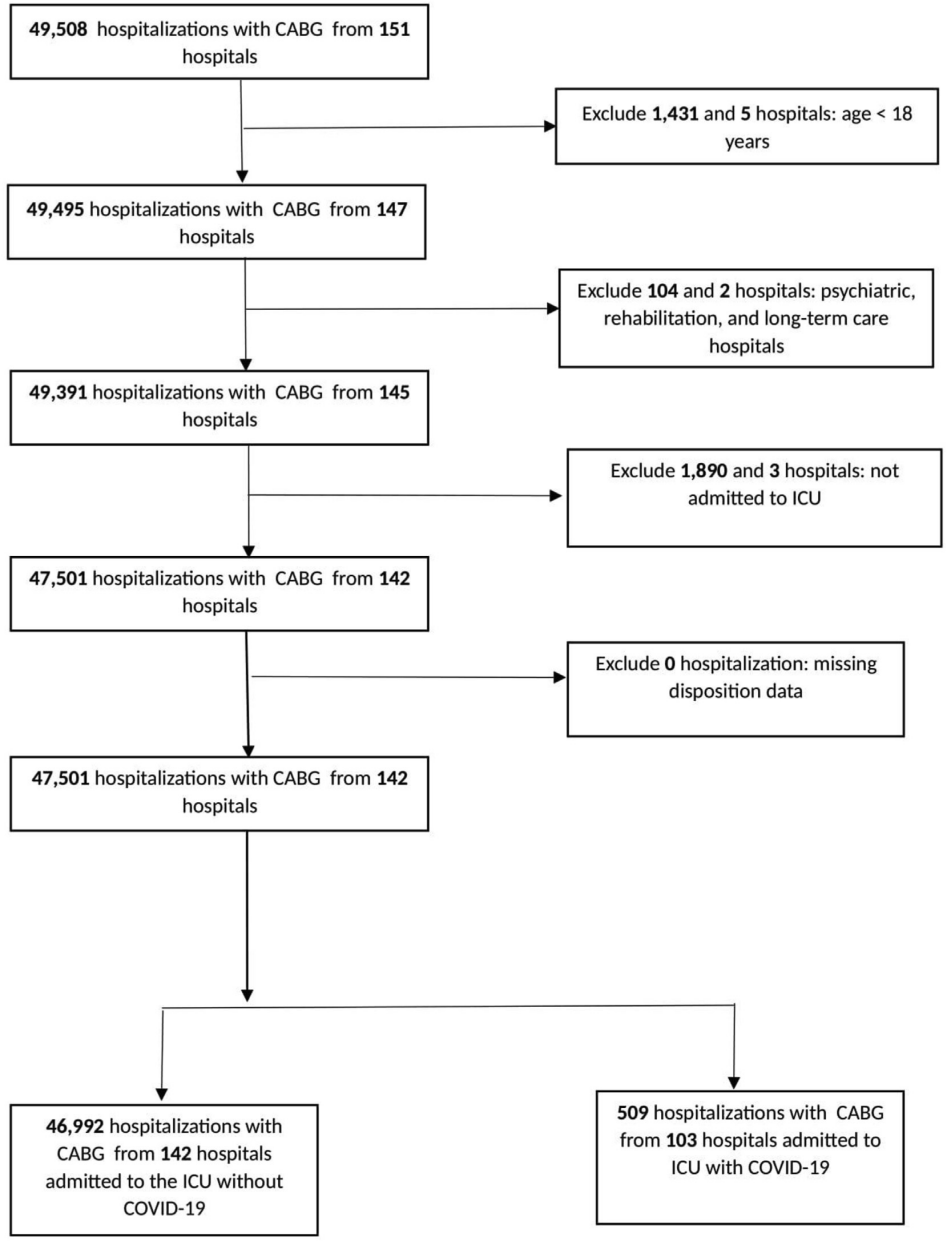
**Cohort derivation.** CABG, coronary artery bypass graft; ICU, intensive care unit.

Our primary cohort consisted of hospitalizations of those aged ≥18 years admitted to the intensive care units (ICU) of acute care hospitals with a procedure code for CABG during the period 2020 Q2 through 2023 Q4. Hospitalizations with missing data on hospital disposition were excluded. We identified hospitalizations with CABG based on the presence of the International Classification of Diseases, Tenth Revision, Procedure Coding System (ICD-10-PCS) codes in any of the surgical procedure columns of the TIPUDF.^11^ ICD-10-PCS codes corresponding to CABG were identified with the aid of the Healthcare Cost and Utilization Project’s Clinical Classification Software Refined (CCSR) for ICD-10-PCS procedures.^11^ CCSR codes incorporate multiple associated ICD-10-PCS codes into clinically meaningful categories. ICD-10-PCS codes corresponding to CABG were selected from CCSR Chapter CAR003 (CABGs) and are listed in Supplemental Table S1. Hospitalizations with ICU admissions were identified based on hospital charges coded for an ICU or coronary care unit. Figure 1 reviews the cohort derivation.

#### Exposure and outcome

The primary exposure was a diagnosis of COVID-19. Hospitalizations with COVID-19 were identified by the presence of the International Classification of Diseases, Tenth Revision, Clinical Modification (ICD-10-CM) codes.^12^ The ICD-10-CM code corresponding to COVID-19, U071, was selected from CCSR for ICD-10-CM diagnosis category INF012: SARS-CoV-2. The primary outcome of in-hospital mortality was identified using code 20 in the PAT_STATUS column of the TIPUDF. Short-term mortality, defined as in-hospital mortality or discharge to a hospice, was used as a secondary outcome with discharge to hospice identified using codes 50 and 51 in the PAT_STATUS column of the TIPUDF.

#### Risk-adjustment covariates

Risk-adjustment covariates were chosen a priori and are based on biological and clinical plausibility. Risk-adjustment covariates include demographics (age, sex, race/ethnicity, type of health insurance), Deyo modification of the Charlson comorbidity index, total number of organ dysfunctions, chronic obstructive pulmonary disease, atrial fibrillation, end-stage renal disease, cardiogenic shock, pulmonary embolism, stroke, systolic heart failure, diastolic heart failure, perivascular disease, sleep apnea, heart block, and acute kidney injury.^13^^,^^14^ Organ dysfunctions were defined using ICD-10-CM codes selected from CSSR categories: RESP012 for respiratory failure; END011 for metabolic failure; GEN002 for renal failure; CIR019 for heart failure; DIG018 for hepatic failure; and NVS011, NVS013, and NVS020 for neurological failure. ICD-10-CM codes for risk adjustment covariates are listed in Supplemental Table S2. Mechanical ventilation and hemodialysis were used as risk-adjustment covariates and identified using ICD-10-PCS codes (Supplemental Table S1). The year of discharge was used as a risk adjustment covariate.

### Statistical analysis

Categorical variables are summarized as counts and percentages. Continuous variables are summarized as mean and standard deviation. The Fisher test was used for the comparison of categorical variables and the *t* test was used for comparison of continuous variables. Two-sided *P* values <.05 were considered statistically significant. Three predefined analysis procedures were applied to examine the association of COVID-19 with in-hospital mortality, short-term mortality, and length of stay: overlap propensity score weights (OW), treated weights, and inverse probability of treatment weights (IPW). OW (the primary analysis approach) was applied for subgroup analysis. Variance inflation factors were examined to exclude multicollinearity in the propensity score model. Analysis results are summarized as adjusted risk ratios (aRR) and 95% CI with adjusted risk difference and 95% CI reported as alternative effect size measures. Balancing weights were applied to estimate average potential outcomes with Hájek estimators.^15^ The aRR and adjusted risk difference were obtained as the ratio and difference of the average potential outcomes and 95% CI were obtained from empirical sandwich variance estimators. The PSweight package was used for each analysis procedure.^15^

#### Propensity score calculation

A propensity score is a probability that indicates the propensity of a CABG hospitalization having COVID-19. The propensity for COVID-19 among CABG hospitalizations was calculated using multilevel, multivariable logistic regression with COVID-19 used as the dependent variable. Individual hospitals were entered as random intercepts to account for clustering within hospitals. All risk-adjustment variables were used in the propensity score calculation.

#### Overlap weights

**Figure 2:**
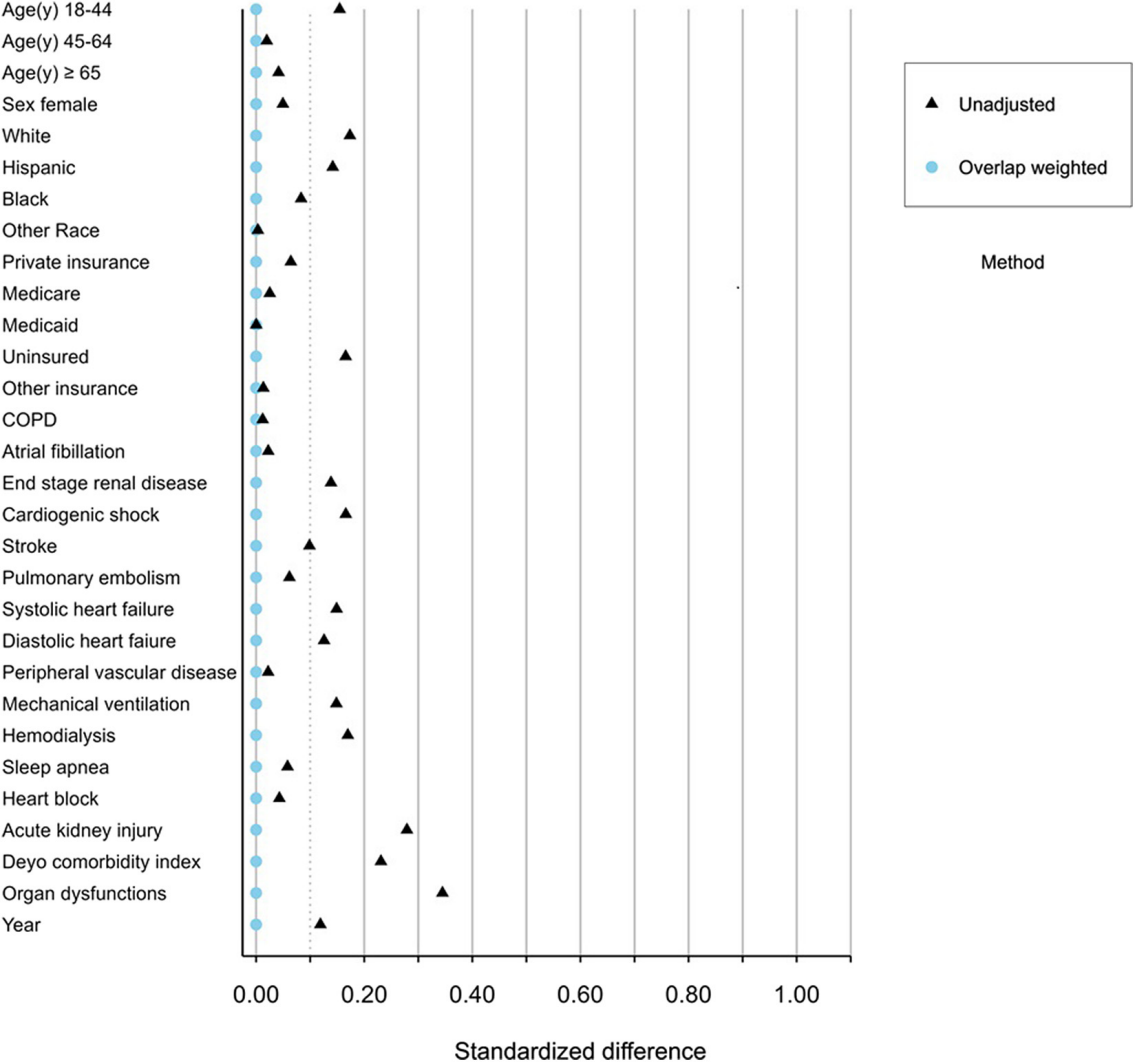
**Standardized difference of means before and after overlap weighting.** COPD, chronic obstructive pulmonary disease.

We used OW to measure the association between COVID-19 and each of the following: in-hospital mortality, short-term mortality, and length of stay in CABG hospitalizations.^16^ For binary exposures, as in this study, the overlap weight of hospitalization is defined as the probability of being in the opposite exposure group: 1 minus the propensity score for hospitalizations with a diagnosis of COVID-19 and for hospitalizations without COVID-19 the propensity score. Overlap weighting results in an exact balance of covariates that are used in the propensity score calculation. Figure 2 displays the absolute standardized difference of means pre and post overlap weighting and Supplemental Table S3 reports the means of covariates within the overlap population. The target estimand for OW is the average treatment effect for the overlap population.

#### Treated weights

We used treated weights to measure the association between COVID-19 and each of the following: in-hospital mortality, short-term mortality, and length of stay in CABG hospitalizations.^17^ The treated weight of a hospitalization with COVID-19 is defined as 1 and the treated weight for a hospitalization without COVID-19 is defined as the propensity score divided by 1 minus the propensity score. The target population for treated weights is the subpopulation with COVID-19 and the target estimand is the average treatment effect for the treated.

#### IPW

We used IPW to measure the association between COVID-19 and each of the following: in-hospital mortality, short-term mortality, and length of stay in CABG hospitalizations.^18^ For binary exposures, the IPW weight is the reciprocal of the probability of a hospitalization being found in the observed treatment group. Thus, for a hospitalization with COVID-19, the IPW weight is the reciprocal of the propensity score, and for a hospitalization without COVID-19, the IPW weight is the reciprocal of 1 minus the propensity score. The target population for IPW is defined as the combined sample and the target estimand is the average treatment effect among the combined population.

#### Subgroup analyses

Additional analyses were performed to evaluate the consistency of the associations between COVID-19 with in-hospital mortality, short-term mortality, and length of stay. Subgroup analyses considered restriction to hospitalizations with community-acquired COVID-19, hospital-acquired COVID-19, ST-elevation myocardial infarction, Non–ST-elevation myocardial infarction, unstable angina, stable angina, hospitalizations without extracorporeal membrane oxygenation, hospitalizations without intraaortic balloon pumps, hospitalizations without prior CABG, hospitalizations without prior percutaneous coronary intervention, year of discharge, non-Hispanic, Hispanic, elective admissions, and nonelective admissions. Nonelective admissions included emergency, urgent, and trauma admissions. The primary analysis approach was used for subgroup analysis. Table 1 and Table 2 report the results of subgroup analyses.

**Table 1:** Subgroup analyses for the association of COVID-19 and mortality in hospitalizations with coronary artery bypass graft.

Subgroup	Mortality		aRR (95% CI)^a^	aRD (95%CI)^b^
	Non–COVID-19	COVID-19		
Community-acquired COVID-19				
In-hospital mortality	1039/46,992 (2.2)	19/355 (5.4)	1.3662 (0.8621-2.1653)	0.0143 (–0.0100 to 0.0385)
Short-term mortality	1157/46,992 (2.5)	23/355 (6.5)	1.4658 (0.9685-2.2186)	0.0207 (–0.0060 to 0.0475)
Hospital-acquired COVID-19				
In-hospital mortality	1039/46,992 (2.2)	13/154 (8.4)	1.8035 (1.0663-3.0502)	0.0399 (–0.0067 to 0.0864)
Short-term mortality	1157/46,992 (2.5)	14/154 (9.1)	1.6997 (1.0268-2.8137)	0.0397 (–0.0085 to 0.0879)
STEMI				
In-hospital mortality	533/16,066 (3.3)	21/292 (7.2)	1.3680 (0.8835-2.1181)	0.0191 (–0.0115 to 0.0496)
Short-term mortality	588/16,066 (3.7)	24/292 (8.2)	1.3779 (0.9182-2.0679)	0.0224 (–0.0102 to 0.0550)
NSTEMI				
In-hospital mortality	399/13,553 (2.9)	13/247 (5.3)	1.1953 (0.6794-2.1028)	0.0084 (–0.0205 to 0.0373)
Short-term mortality	443/13,553 (3.3)	16/247 (6.5)	1.2963 (0.7836-2.1445)	0.0147 (–0.0174 to 0.0468)
Unstable angina				
In-hospital mortality	220/13,738 (1.6)	5/160 (3.1)	1.5021 (0.6240-3.6163)	0.0109 (–0.0175 to 0.0393)
Short-term mortality	239/13,738 (1.7)	6/160 (3.8)	1.5748 (0.7070-3.5077)	0.0143 (–0.0167 to 0.0454)
Stable angina				
In-hospital mortality	819/33,254 (2.5)	27/349 (7.7)	1.5659 (1.0690-2.2937)	0.0284 (–0.0010 to 0.0579)
Short-term mortality	918/33,254 (2.8)	31/349 (8.9)	1.5678 (1.1013-2.2319)	0.0329 (0.0014-0.0644)
No ECMO				
In-hospital mortality	925/46,790 (2.0)	28/503 (5.6)	1.5427 (1.0569-2.2517)	0.0198 (−0.0011 to 0.0408)
Short-term mortality	1038/46,790 (2.2)	33/503 (6.6)	1.5735 (1.1136-2.2232)	0.0244 (0.0017-0.0471)
No IABP				
In-hospital mortality	873/45,602 (1.9)	28/484 (5.8)	1.6301 (1.1173-2.3783)	0.0227 (0.0009-0.0444)
Short-term mortality	976/45,602 (2.1)	32/484 (6.6)	1.6232 (1.1429-2.3054)	0.0259 (0.0027-0.0491)
No PCAB				
In-hospital mortality	998/44,062 (2.3)	32/490 (6.5)	1.5610 (1.0989-2.2175)	0.0239 (0.0010-0.0468)
Short-term mortality	1113/44,062 (2.5)	37/490 (7.6)	1.5861 (1.1471-2.1931)	0.0285 (0.0040-0.0531)
No PPCI				
In-hospital mortality	956/40,922 (2.3)	30/459 (6.5)	1.5536 (1.0812-2.2323)	0.0238 (0.0000-0.0476)
Short-term mortality	1064/40,922 (2.6)	35/459 (7.6)	1.5959 (1.1440-2.2265)	0.0293 (0.0036-0.0549)
Year 2020				
In-hospital mortality	209/8472 (2.5)	8/53 (15.1)	3.4244 (1.7558-6.6788)	0.1083 (0.0102-0.2064)
Short-term mortality	224/8472 (2.6)	8/53 (15.1)	3.2402 (1.6650-6.3060)	0.1058 (0.0077-0.2038)
Year 2021				
In-hospital mortality	286/12,437 (2.3)	13/114 (11.4)	2.7480 (1.5953-4.7335)	0.0753 (0.0142-0.1363)
Short-term mortality	321/12,437 (2.6)	15/114 (13.2)	2.5331 (1.5305-4.1923)	0.0822 (0.0174-0.1470)
Year 2022				
In-hospital mortality	289/12,731 (2.3)	9/229 (3.9)	0.8436 (0.4204-1.6927)	–0.0068 (–0.0325 to 0.0189)
Short-term mortality	326/12,731 (2.6)	12/229 (5.2)	1.0198 (0.5641-1.8436)	0.0010 (–0.0291 to 0.0311)
Year 2023				
In-hospital mortality	255/13,352 (1.9)	2/113 (1.8)	0.5858 (0.1464-2.3437)	–0.0130 (–0.0391 to 0.0130)
Short-term mortality	286/13,352 (2.1)	2/113 (1.8)	0.5005 (0.1253-1.9993)	–0.0184 (–0.0446 to 0.0078)
Non-Hispanic				
In-hospital mortality	755/36,152 (2.1)	24/360 (6.7)	1.5813 (1.0516-2.3777)	0.0244 (–0.0022 to 0.0509)
Short-term mortality	854/36,152 (2.4)	28/360 (7.8)	1.5896 (1.0933-2.3110)	0.0289 (0.0003-0.0575)
Hispanic				
In-hospital mortality	284/10,838 (2.6)	8/149 (5.4)	1.5151 (0.7620-3.0126)	0.0198 (–0.0197 to 0.0592)
Short-term mortality	303/10,838 (2.8)	9/149 (6.0)	1.5748 (0.8253-3.0051)	0.0239 (–0.0178 to 0.0655)
Elective admissions				
In-hospital mortality	333/24,247 (1.4)	6/147 (4.1)	2.0691 (0.9343-4.5824)	0.0227 (–0.0118 to 0.0572)
Short-term mortality	375/24,247 (1.5)	7/147 (4.8)	2.0759 (0.9971-4.3219)	0.0266 (–0.0106 to 0.0637)
Nonelective admission				
In-hospital mortality	706/22,745 (3.1)	26/362 (7.2)	1.4187 (0.9603-2.0959)	0.0211 (–0.0064 to 0.0487)
Short-term mortality	782/22,745 (3.4)	30/362 (8.3)	1.4357 (1.0011-2.0591)	0.0252 (–0.0043 to 0.0547)

Values are n/N (%) unless noted.

aRD, adjusted risk difference; aRR, adjusted risk ratio; ECMO, extracorporeal membrane oxygenation; IABP, intra-aortic balloon pump; NSTEMI, Non–ST-elevation myocardial infarction; PCAB, prior coronary artery bypass graft; PPCI, prior percutaneous coronary intervention; STEMI, ST-elevation myocardial infarction.

**Table 2:** Subgroup analyses for the association of COVID-19 and length of stay in hospitalizations with coronary artery bypass graft.

Subgroup	Length of stay, d		aRR (95% CI)	aRD (95%CI)
	Non–COVID-19	COVID-19		
Community-acquired COVID-19	9.62 ± 6.79	15.08 ± 11.48	1.3691 (1.2596-1.4881)	4.0858 (2.8288-5.3428)
Hospital-acquired COVID-19	9.62 ± 6.79	18.70 ± 11.45	1.4851 (1.3412-1.6445)	6.0280 (4.1568-7.8993)
ST-elevation myocardial infarction	11.74 ± 7.26	17.03 ± 10.68	1.3130 (1.2165-1.4171)	4.0659 (2.7765-5.3553)
Non–ST-elevation myocardial infarction	11.86 ± 7.30	17.21 ± 10.97	1.3314 (1.2234-1.4490)	4.2921 (2.8457-5.7384)
Unstable angina	9.43 ± 6.36	14.40 ± 8.40	1.3662 (1.2414-1.5036)	3.8421 (2.4791-5.2051)
Stable angina	9.69 ± 6.96	16.99 ± 12.70	1.4235 (1.3101-1.5467)	5.0507 (3.6552-6.4462)
No extracorporeal membrane oxygenation	9.56 ± 6.58	15.65 ± 9.81	1.3745 (1.2971-1.4566)	4.2457 (3.3510-5.1403)
No intraaortic balloon pump	9.47 ± 6.55	15.99 ± 11.17	1.4118 (1.3225-1.5072)	4.6451 (3.6130-5.6772)
No prior coronary artery bypass graft	9.62 ± 6.90	16.28 ± 11.74	1.4046 (1.3131-1.5026)	4.6701 (3.5863-5.7540)
No prior percutaneous coronary intervention	9.76 ± 6.82	16.48 ± 11.79	1.3997 (1.3060-1.5001)	4.6834 (3.5566-5.8102)
Year 2020	9.40 ± 7.55	17.30 ± 11.62	1.5768 (1.3092-1.8991)	6.2082 (3.0889-9.3275)
Year 2021	9.54 ± 6.52	17.54 ± 15.80	1.5546 (1.3077-1.8481)	6.2874 (3.2605-9.3142)
Year 2022	9.65 ± 6.59	15.83 ± 9.77	1.3587 (1.2481-1.4791)	4.1450 (2.8351-5.4550)
Year 2023	9.79 ± 6.72	14.99 ± 9.75	1.3205 (1.1631-1.4992)	3.6364 (1.7464-5.5263)
Hispanic	10.04 ± 6.75	16.70 ± 11.28	1.4154 (1.2613-1.5883)	4.9342 (3.0154-6.8529)
Non-Hispanic	9.49 ± 6.80	15.96 ± 11.70	1.4072 (1.2991-1.5244)	4.5930 (3.3347-5.8512)
Elective admissions	7.64 ± 5.08	12.63 ± 9.82	1.4279 (1.2498-1.6314)	3.7203 (2.0727-5.3678)
Nonelective admission	11.72 ± 7.69	17.62 ± 11.93	1.3215 (1.2284-1.4217)	4.2919 (3.0134-5.5703)

Values are mean ± SD unless noted.

aRD, adjusted risk difference; aRR, adjusted risk ratio.

#### Sensitivity analyses

To address unmeasured confounding, E-values were computed to determine the magnitude of association that unmeasured confounders must have to negate the results of the primary model.^19^ An E-value represents the minimum strength of association, on the risk ratio scale, that unmeasured confounders would need with both the exposure and outcome, conditioned on the measured risk adjustment covariates, to fully explain away an association. A large E-value implies that substantial unmeasured confounding is required to explain away the effect estimate. E-values for the point estimate and the lower bound of the 95% CI are reported in Table 3.

**Table 3:** E-values for association of COVID-19 and outcomes in hospitalizations with CABG.

Outcome	aRR (E-value)	95% CI lower bound (E-Value)
In-hospital mortality	1.5394 (2.4506)	1.0836 (1.3846)
Short-term mortality	1.5635 (2.5021)	1.1306 (1.5149)
Length of stay	1.4028 (2.1545)	1.3138 (1.9556)

aRR, adjusted risk ratio; CABG, coronary artery bypass graft.

#### Software and data management

Data management used Excel (Microsoft) and statistical analyses used R 4.4.1 (R Foundation for Statistical Computing). The R code used for this study is provided in the Supplemental Material.

## Results

We identified 47,501 hospitalizations with a procedure code for CABG, of which 509 (1.1%) had COVID-19. Figure 1 reviews the cohort derivation. Propensity score overlap weighting resulted in the exact balance of all covariates (Supplemental Table S3). The absolute standardized difference of means of the weighted and unweighted groups is compared in Figure 2.

### Cohort characteristics

The characteristics of CABG hospitalizations with vs without COVID-19 are summarized in Table 4 and Table 5. CABG hospitalizations with vs without COVID-19 had higher comorbidity index (2.01 ± 1.65 vs 1.65 ± 1.61), more frequent need for invasive mechanical ventilation (12.8% vs 8.5%), had higher rates of myocardial infarction (65.0% vs 45.6%), higher rates of congestive heart failure (53.4% vs 40.1%), and higher rates of acute kidney injury (35.6% vs 24.3%); *P* < .0001 for each comparison.

**Table 4:** The demographic and administrative characteristics of critically ill CABG hospitalizations with and without COVID-19.

Variable	Non–COVID-19 (n = 46,992)	COVID-19 (n = 509)	SMD	*P* value
Age, y			0.088	.0003
18-44	1228 (2.6)	31 (6.1)	–	–
45-64	18,615 (39.6)	197 (38.7)	–	–
≥65	27,149 (57.8)	281 (55.2)	–	–
Sex^a^			0.0505	.2871
Male	33,272 (75.3)	371 (77.5)	–	–
Female	10,910 (24.7)	108 (22.5)	–	–
Race/ethnicity^b^			0.1467	.0013
White	28,670 (61.0)	268 (52.7)	–	–
Hispanic	10,838 (23.1)	149 (29.3)	–	–
Black	3376 (7.2)	45 (8.8)	–	–
Other	4106 (8.7)	47 (9.2)	–	–
Health insurance^c^			0.1619	0.0079
Private	25,777 (55.1)	261 (51.5)	–	–
Medicare	15,280 (32.7)	161 (31.8)	–	–
Medicaid	1383 (3.0)	16 (3.2)	–	–
Uninsured	3143 (6.7)	56 (11.0)	–	–
Other	1192 (2.5)	13 (2.6)	–	–
Teaching hospital	14,155 (30.1)	189 (37.1)	0.1488	.0008
Length of stay, d	9.62 ± 6.79	16.18 ± 11.58	0.9564	<.0001
Hospital disposition			0.5450	.0001
Hospital mortality	1039 (2.2)	32 (6.3)	–	–
Another acute care hospital	334 (0.7)	9 (1.8)	–	–
Hospice	118 (0.3)	5 (1.0)	–	–
Home self-care	26,857 (57.2)	234 (46.0)	–	–
Home skilled care	9821 (20.9)	89 (17.5)	–	–
Chronic care facility^d^	8607 (18.3)	139 (27.3)	–	–
Leave against medical advice	81 (0.2)	0 (0)	–	–
Other	135 (0.3)	1 (0.2)	–	–

Values are n (%) or mean ± SD.

CABG, coronary artery bypass graft; SMD, standardized mean difference.

a Sex data were missing for 2810 hospitalizations without and 30 hospitalizations with COVID-19.

b Race/ethnicity information was missing for 2 hospitalizations without COVID-19.

c Health insurance data were missing for 217 hospitalizations without and 2 hospitalizations with COVID-19.

d Chronic care facilities include long-term care hospitals, inpatient rehabilitation, skilled nursing facilities, and nursing homes.

**Table 5:** Comorbidities, complications, and procedures of critically ill CABG hospitalizations with and without COVID-19.

Variable	Non–COVID-19 (n = 46,992)	COVID-19 (n = 509)	SMD	*P* value
Deyo comorbidity index	1.65 ± 1.61	2.01 ± 1.65	0.2235	<.0001
Deyo-Charlson comorbidities				
Myocardial infarction	21,443 (45.6)	331 (65.0)	0.3978	<.0001
Congestive heart failure	18,830 (40.1)	272 (53.4)	0.2704	<.0001
Peripheral vascular disease	4849 (10.3)	46 (9.0)	0.0434	.3792
Cerebrovascular disease	5148 (11.0)	64 (12.6)	0.0503	.2535
Dementia	636 (1.4)	12 (2.4)	0.0745	.0782
Chronic lung disease	8430 (17.9)	89 (17.5)	0.0119	.8616
Rheumatological disease	937 (2.0)	23 (4.5)	0.1426	.0004
Peptic ulcer disease	368 (0.8)	6 (1.2)	0.0402	.3036
Mild liver disease	1339 (2.8)	19 (3.7)	0.0495	.2275
Moderate/severe liver disease	95 (0.2)	2 (0.4)	0.0350	.2788
Diabetes without complications	19,766 (42.1)	221 (43.4)	0.0274	.5574
Diabetes with complications	11,887 (25.3)	135 (26.5)	0.0280	.5385
Hemiplegia or paraplegia	459 (1.0)	6 (1.2)	0.0196	.6455
Renal disease	10,629 (22.6)	130 (25.5)	0.0684	.1225
Malignancy	982 (2.1)	8 (1.6)	0.0386	.5315
Metastatic solid tumor	106 (0.2)	1 (0.2)	0.0063	1.0000
Human immunodeficiency virus	82 (0.2)	0 (0.0)	0.0591	1.0000
Other comorbidities				
Atrial fibrillation	14,964 (31.8)	155 (30.5)	0.0301	.5341
Chronic kidney disease	5966 (12.7)	65 (12.8)	0.0022	.9466
Cardiogenic shock	6276 (13.4)	100 (19.6)	0.1701	.0001
Stroke	986 (2.1)	18 (3.5)	0.0870	.0413
Pulmonary embolism	620 (1.3)	10 (2.0)	0.0508	.2356
Rejection fraction	7662 (16.3)	115 (22.6)	0.1594	.0002
Heart block	1720 (3.7)	24 (4.7)	0.0527	.1932
Acute kidney injury	11,417 (24.3)	181 (35.6)	0.2479	<.0001
Chronic obstructive pulmonary disease	5638 (12.0)	62 (12.2)	0.0056	.8909
Hypothyroidism	5763 (12.3)	55 (10.8)	0.0457	.3417
Liver disease	115 (0.2)	3 (0.6)	0.0535	.1337
Perivascular disease	1634 (3.5)	16 (3.1)	0.0187	.8074
Sleep apnea	5824 (12.4)	53 (10.4)	0.0624	.1982
Number of organ dysfunctions	1.15 ± 1.22	1.60 ± 1.48	0.3679	<.0001
Types of organ dysfunctions				
Respiratory	11,729 (25.0)	213 (41.8)	0.3639	<.0001
Cardiovascular	12,227 (26.0)	150 (29.5)	0.0771	.0842
Renal	11,417 (24.3)	181 (35.6)	0.2479	<.0001
Cardiovascular	778 (1.7)	17 (3.3)	0.1081	.0078
Hepatic	10,919 (23.2)	120 (23.6)	0.0080	.8742
Hematological	3977 (8.5)	60 (11.8)	0.1104	.0103
Neurological	2905 (6.2)	72 (14.1)	0.2659	<.0001
Medical procedures				
Mechanical ventilation	4006 (8.5)	65 (12.8)	0.1380	.0014
Hemodialysis	1413 (3.0)	33 (6.5)	0.1641	.0001
Blood transfusion	8190 (17.4)	112 (22.0)	0.1152	.0082
Electrical cardioversion	1064 (2.3)	15 (2.9)	0.0429	.2929
Echocardiography	12,541 (26.7)	119 (23.4)	0.0764	.0963
Temporary mechanical support devices	1604 (3.4)	30 (5.9)	0.1180	.0045
Right heart catheterization	650 (1.4)	15 (2.9)	0.1076	.0067
Heart failures and complications				
ST-elevation myocardial infarction	16,066 (34.2)	292 (57.4)	0.4784	<.0001
Non–ST-elevation myocardial infarction	13,553 (28.8)	247 (48.5)	0.4127	<.0001
Diastolic heart failure	4313 (9.2)	64 (12.6)	0.1092	.0109
Systolic heart failure	7662 (16.3)	115 (22.6)	0.1594	.0002
Uncomplicated hypertension	21,413 (45.6)	170 (33.4)	0.2509	<.0001
Complicated hypertension	21,669 (46.1)	277 (54.4)	0.1667	.0002
Prior coronary artery bypass graft	2930 (6.2)	19 (3.7)	0.1152	.0163
Prior percutaneous coronary intervention	6070 (12.9)	50 (9.8)	0.0976	.0390
Prior myocardial infarction	8130 (17.3)	78 (15.3)	0.0535	.2627
Intraaortic balloon pump	1390 (3.0)	25 (4.9)	0.1006	.0171
Extracorporeal membrane oxygenation				
Assistance with respiratory filtration, continuous	0 (0.0)	0 (0.0)	0.0000	1.0000
Extracorporeal membrane oxygenation, continuous	0 (0.0)	0 (0.0)	0.0000	1.0000
Extracorporeal oxygenation, membrane, central	32 (0.1)	1 (0.2)	0.0353	.2993
Extracorporeal oxygenation, membrane, peripheral veno-arterial	152 (0.3)	4 (0.8)	0.0623	.0876
Extracorporeal oxygenation, membrane, peripheral veno-venous	32 (0.1)	2 (0.4)	0.0678	.0513

Values are n (%) or mean ± SD.

CABG, coronary artery bypass graft; SMD, standardized mean difference.

### Association of COVID-19 and mortality

The dispositions of CABG hospitalizations with vs without COVID-19 are provided in Table 4. CABG hospitalizations with COVID-19 had higher unadjusted in-hospital mortality, compared to those without COVID-19 (6.3*%* vs 2.2%). The increased risk of in-hospital mortality among CABG hospitalizations with COVID-19 remained in adjusted analysis using the primary model, being 53.9% higher (aRR, 1.5394; 95% CI, 1.0836 to 2.1870). Both alternative modeling analyses produced similar results to the primary model (Table 6). In subgroup analysis (Table 1), the association of COVID-19 and in-hospital mortality was mostly consistent across the examined subgroups with the notable exception of year. The association of COVID-19 with in-hospital mortality was highest during the first year of the pandemic (aRR, 3.4244; 95% CI, 1.7558 to 6.6788) and decreased year over year with no association observed in 2023 (aRR, 0.5858; 95% CI, 0.1464 to 2.3437).

**Table 6:** Association of COVID-19 and outcomes in hospitalizations with coronary artery bypass graft.

Outcome	aRR (95% CI)	aRD (95% CI)
In-hospital mortality		
Overlap weights	1.5394 (1.0836-2.1870)	0.0224 (0.0003-0.0446)
Treated weights	1.5343 (1.0798-2.1800)	0.0226 (0.0002-0.0451)
Inverse probability weights	1.7303 (1.1372-2.6325)	0.0163 (0.0002-0.0325)
Short-term mortality		
Overlap weights	1.5635 (1.1306-2.1622)	0.0268 (0.0031-0.0505)
Treated weights	1.5569 (1.1257-2.1533)	0.0270 (0.0030-0.0510)
Inverse probability weights	1.7630 (1.1950-2.6009)	0.0191 (0.0021-0.0361)
Length of stay, d		
Overlap weights	1.4028 (1.3138-1.4978)	4.6328 (3.5838-5.6817)
Treated weights	1.4008 (1.3110-1.4966)	4.6345 (3.5718-5.6973)
Inverse probability weights	1.4270 (1.3443-1.5148)	4.0900 (3.2769-4.9032)

aRD, adjusted risk difference; aRR, adjusted risk ratio.

### Association of COVID-19 and length of stay

CABG hospitalizations with COVID-19 had a higher unadjusted length of stay, compared to those without COVID-19 (16.2 ± 11.6 days vs 9.6 ± 6.8 days). The longer length of stay among CABG hospitalizations with COVID-19 remained in adjusted analysis using the primary model, being 40.3% higher (aRR, 1.4028; 95% CI, 1.3138 to 1.4978). Both alternative modeling procedures produced similar results to the primary model (Table 6). In subgroup analysis, COVID-19 was consistently associated with increased length of stay across all examined subgroups including individual years (Table 2, Central Illustration). In contrast to mortality, length of stay remained strongly associated with COVID-19 during 2023 (aRR, 1.3205; 95% CI, 1.1631 to 1.4992).

**Central Illustration:**
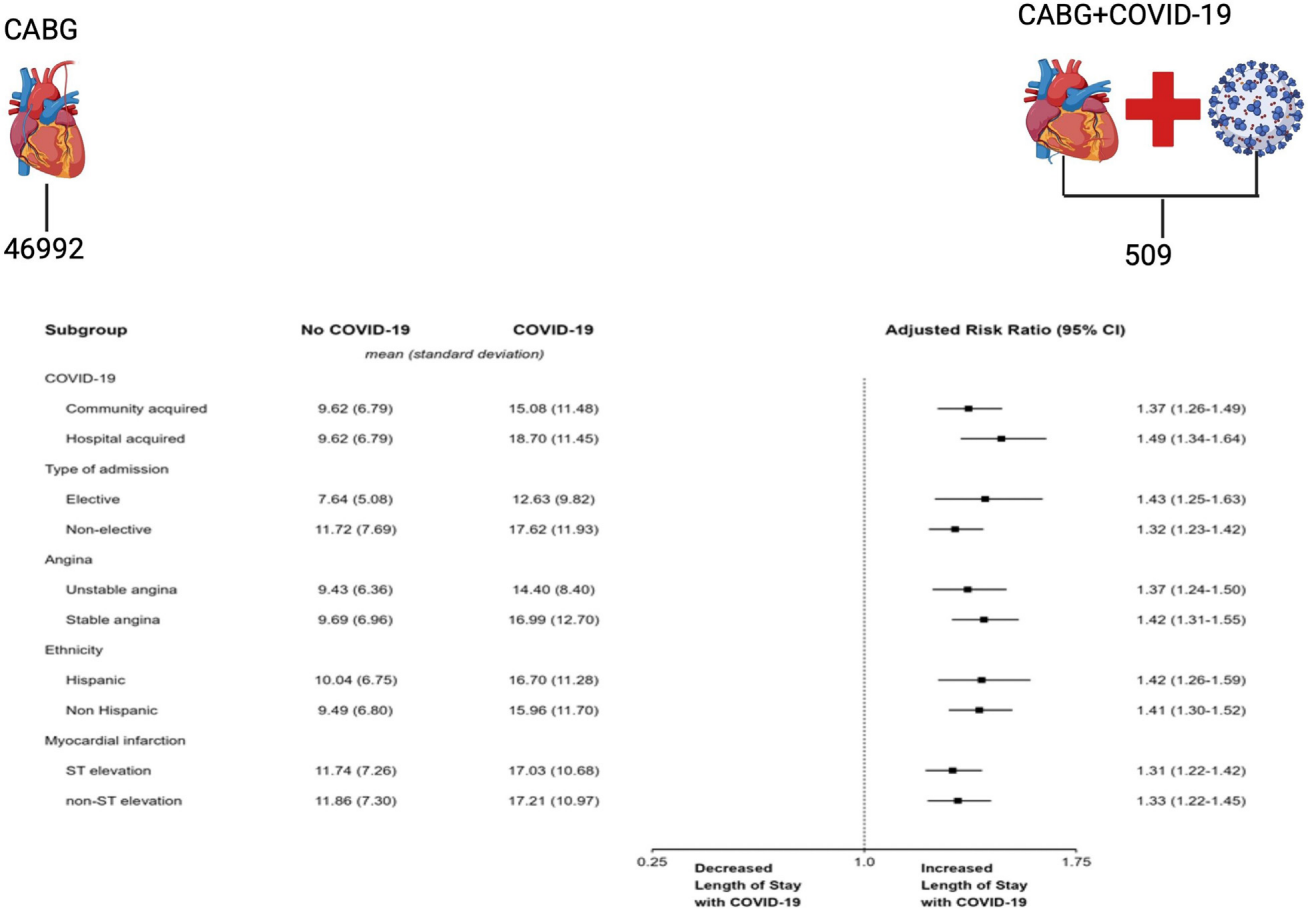
Strength of association of length of stay and COVID-19. CABG, coronary artery bypass graft.

### Sensitivity analyses for unmeasured confounders

The E-values for the point estimates and lower bound of 95% CI of aRR reported in Table 6 are presented in Table 3. To fully explain away the observed association between in-hospital mortality and COVID-19, an unmeasured confounder would have to be associated with both COVID-19 and in-hospital mortality at the level 2.4506 on the risk ratio scale. Similarly, associations at levels 2.501 and 2.1545 on the risk ratio scale would be needed to fully explain away the measured association of COVID-19 with short-term mortality and length of stay, respectively.

## Discussion

### Key findings

In this population-based cohort study, among hospitalizations with CABG, a diagnosis of COVID-19 was associated with 53.9% higher risk of in-hospital mortality, 56.4% higher risk of short-term mortality, and a 40.3% longer length of stay. This finding was robust across 3 predefined estimation methods and in subgroup analyses. Hospitalizations with a diagnosis of COVID-19 were also observed to constitute a distinctive group with respect to baseline demographic and medical characteristics. Notably, hospitalized patients with a diagnosis of COVID-19 were younger and more often Hispanic. They also had higher mean Deyo comorbidity index, higher mean number of organ dysfunctions, higher rates of myocardial infarction, congestive heart failure, rheumatological disease, cardiogenic shock, and rejection fraction. Hospitalizations with COVID-19 also demonstrated higher rates of medical procedures; notably invasive mechanical ventilation, hemodialysis, blood transfusions, temporary mechanical support devices, and right-heart catheterization were each more frequent in hospitalizations with COVID-19.

### Relations to previous studies

COVID-19 significantly compromises the cardiovascular system. According to a comprehensive meta-analysis involving 1527 patients, 8% of COVID-19 patients also had concomitant heart damage.^7^ An increasing amount of data indicate that serious myocardial injury may result from endothelial dysfunction via the angiotensin-converting enzyme 2 receptor. In cases of severe disease, the SARS-CoV-2–associated systemic inflammatory response syndrome induces a cytokine release syndrome that may damage cardiac myocytes and vascular endothelium. Myocardial damage is linked to the higher cardiometabolic demand from systemic inflammation and the persistent hypoxia brought on by pneumonia or acute respiratory distress syndrome.^7^^,^^20^ The process mentioned above contributes to the morbidity and mortality seen in COVID-19 patients undergoing CABG and cardiac surgeries in general. Our study showed that patients who had COVID-19 while undergoing CABG had increased mortality as opposed to those without a diagnosis of COVID-19, which was statistically significant after adjusting for other comorbidities (Table 1). Previous studies have demonstrated that arrhythmias, sudden cardiac arrest, new-onset cardiomyopathy, cardiogenic shock, and heart failure are more often reported in these patients.^21^, ^22^, ^23^ Regarding arrhythmias, with an incidence of 15% to 45%, atrial fibrillation is the most common atrial arrhythmia after CABG, which arises due to physical trauma, mechanical stress, and myocardial inflammation (caused by the infection).^24^^,^^25^ Our study did not show any statistically significant difference in the rate of atrial fibrillation between groups (Table 5).

The mean length of stay in our study for COVID-19 patients was 16.2 days and 9.6 days in non–COVID-19 patients; following adjusted analysis, a 40.3% statistically significant increase in length of stay in the COVID-19 group was measured (Table 6). In patients without COVID-19, a similar mean length of stay was seen in a study by Agarwal et al^8^; however, the length of stay was higher in patients who had emergency CABG. Our study had a higher mean length of stay in the COVID-19 group of 16.2 days as opposed to the average in the study, as mentioned earlier, which was 12.5 days.^8^ In subgroup analyses for emergent and urgent CABG, there were higher rates of short-term mortality and longer mean length of stay which was slightly comparable to the result of a study by Keskin et al,^26^ where COVID-19 patients had only a significant increase in prolonged mechanical ventilation and length of stay for CABG within 1 day of infection and no positive correlation was seen in a similar subset that had CABG within 30 days of the disease. Further subgroup analysis showed no significant difference in in-hospital mortality in patients with emergency CABG. For elective CABG, there was no significant difference in both in-hospital and short-term mortality; however, there was an increase in length of stay. Also, for patients who had CABG during the pandemic (2020 and 2021), there was an increase in both short-term and in-hospital mortalities, which was not seen during subsequent years prior to emerging therapies used to reduce morbidity and mortality associated with the infection. The mean length of stay has increased in COVID-19 patients since the onset of the disease.^8^^,^^26^ Factors that have been previously associated with increased length of stay include mechanical ventilation and complications like pneumonia, reexploration, acute respiratory distress syndrome, renal dysfunction, stroke, pulmonary embolism, pericardial effusion, and atrial fibrillation.^27^^,^^28^ In our study, many similar factors such as acute kidney injury, mechanical ventilation, temporary mechanical support devices, blood transfusions, mechanical ventilation, heart failure with reduced ejection fraction, stroke, and cardiogenic shock were demonstrated to be significantly increased in the COVID-19 group (Table 5). In contrast, the difference in rates of pulmonary embolism was not statistically significant.

### Study strengths and limitations

This study has several relative strengths and limitations. Regarding strengths, we used a large population-level data set covering the entirety of an expansive and diverse region over an extended period, allowing for the transcendence of local practices and a mix of patient characteristics. The study also used a wide collection of risk adjustment covariates that included demographics, medical procedures, and diagnoses. We used 3 prespecified analysis procedures each based on a nonparametric estimator that did not require any outcome model specification. An extensive subgroup analysis has been completed to review the consistency of the association of COVID-19 with mortality and length of stay across several hospitalization strata. We have computed E-values to determine the minimum association that unmeasured confounders must have to explain away the reported associations. The study has, however, important limitations mostly due to the use of administrative data and the number of CABG hospitalizations with COVID-19. First, the size of the exposure group made more nuanced subgroup analyses impractical so that we could not determine the consistency of the association of COVID-19 and mortality across certain demographic and risk strata such as Blacks and hospitalizations with intraaortic balloon pump or extracorporeal membrane oxygenation. Secondly, we could not account for repeated hospital admissions in the data which could have influenced the results. It should be noted, however, that other epidemiological studies in the US (for example those using the National Inpatient Sample) face similar limitations. Thirdly, we could not determine vaccination status or particular strains of COVID-19. Fourth, for hospital-acquired COVID-19, we could not determine the chronological order or duration between COVID-19 infection and CABG. Fifthly, we could not determine the age of grafts or the specific techniques used for CABG. Sixth, residual confounding for unaccounted variables is possible. Lastly, the generalizability of our findings to other geographic areas is not known.

## Conclusion

COVID-19 was associated with a significantly higher risk of in-hospital and short-term mortality in CABG hospitalizations during the study period. However, the association was most significant at the beginning of the pandemic and decreased until it vanished in 2023. Longer length of stay was strongly associated with a diagnosis of COVID-19 in hospitalizations with CABG and this association has remained through 2023. Additional studies can develop an understanding of the long-term impacts of COVID-19 on CABG patients.

## Footnotes

To access the supplementary material accompanying this article, visit the online version of the *Journal of the Society for Cardiovascular Angiography & Interventions* at 10.1016/j.jscai.2025.103605.
