# Supplementary material for: The Association of COVID-19 and Mortality in Hospitalizations With Coronary Artery Bypass Graft
Source: J Soc Cardiovasc Angiogr Interv. 2025 May 13;4(8):103605. doi: 10.1016/j.jscai.2025.103605 (PMC12462153; doi:10.1016/j.jscai.2025.103605)
Supplement: Supplementary Material [file mmc1.docx]

**The Association of COVID-19 and Mortality in Hospitalizations with Coronary Artery Bypass Grafting**

**Supplementary Materials**

Godfrey Tabowei MD, John Garza PhD, Fath Ayman MD, Ahmed Bashir Sukhera MD, Gabriel Alugba MD, Oboseh John Ogedegbe MD, Samuel Dadzie MD, Ooreoluwa Fasola MD, Meron Tesfaye MS4, Evbu Enakpene MD, Prasad Anand MD

| **Supplementary Table S1. *International Classification of Diseases, Tenth Revisions, Procedure Coding System* (ICD-10-PCS) Codes** | |
| --- | --- |
| **Procedures** | **ICD-10-PCS codes** |
| Coronary artery bypass grafts | 0210083, 0210088, 0210089, 021008C, 021008F, 021008W, 0210093, 0210098, 0210099, 021009C, |
|  | 021009F, 021009W, 02100A3, 02100A8, 02100A9, 02100AC, 02100AF, 02100AW, 02100J3, 02100J8, |
|  | 02100J9, 02100JC, 02100JF, 02100JW, 02100K3, 02100K8, 02100K9, 02100KC, 02100KF, 02100KW, |
|  | 02100Z3, 02100Z8, 02100Z9, 02100ZC, 02100ZF, 0210444, 0210483, 0210488, 0210489, 021048C, |
|  | 021048F, 021048W, 0210493, 0210498, 0210499, 021049C, 021049F, 021049W, 02104A3, 02104A8, |
|  | 02104A9, 02104AC, 02104AF, 02104AW, 02104D4, 02104J3, 02104J8, 02104J9, 02104JC, 02104JF, |
|  | 02104JW, 02104K3, 02104K8, 02104K9, 02104KC, 02104KF, 02104KW, 02104Z3, 02104Z8, 02104Z9, |
|  | 02104ZC, 02104ZF, 0211083, 0211088, 0211089, 021108C, 021108F, 021108W, 0211093, 0211098, |
|  | 0211099, 021109C, 021109F, 021109W, 02110A3, 02110A8, 02110A9, 02110AC, 02110AF, 02110AW, |
|  | 02110J3, 02110J8, 02110J9, 02110JC, 02110JF, 02110JW, 02110K3, 02110K8, 02110K9, 02110KC, |
|  | 02110KF, 02110KW, 02110Z3, 02110Z8, 02110Z9, 02110ZC, 02110ZF, 0211444, 0211483, 0211488, |
|  | 0211489, 021148C, 021148F, 021148W, 0211493, 0211498, 0211499, 021149C, 021149F, 021149W, |
|  | 02114A3, 02114A8, 02114A9, 02114AC, 02114AF, 02114AW, 02114D4, 02114J3, 02114J8, 02114J9, |
|  | 02114JC, 02114JF, 02114JW, 02114K3, 02114K8, 02114K9, 02114KC, 02114KF, 02114KW, 02114Z3, |
|  | 02114Z8, 02114Z9, 02114ZC, 02114ZF, 0212083, 0212088, 0212089, 021208C, 021208F, 021208W, |
|  | 0212093, 0212098, 0212099, 021209C, 021209F, 021209W, 02120A3, 02120A8, 02120A9, 02120AC, |
|  | 02120AF, 02120AW, 02120J3, 02120J8, 02120J9, 02120JC, 02120JF, 02120JW, 02120K3, 02120K8, |
|  | 02120K9, 02120KC, 02120KF, 02120KW, 02120Z3, 02120Z8, 02120Z9, 02120ZC, 02120ZF, 0212444, |
|  | 0212483, 0212488, 0212489, 021248C, 021248F, 021248W, 0212493, 0212498, 0212499, 021249C, |
|  | 021249F, 021249W, 02124A3, 02124A8, 02124A9, 02124AC, 02124AF, 02124AW, 02124D4, 02124J3, |
|  | 02124J8, 02124J9, 02124JC, 02124JF, 02124JW, 02124K3, 02124K8, 02124K9, 02124KC, 02124KF, |
|  | 02124KW, 02124Z3, 02124Z8, 02124Z9, 02124ZC, 02124ZF, 0213083, 0213088, 0213089, 021308C, |
|  | 021308F, 021308W, 0213093, 0213098, 0213099, 021309C, 021309F, 021309W, 02130A3, 02130A8, |
|  | 02130A9, 02130AC, 02130AF, 02130AW, 02130J3, 02130J8, 02130J9, 02130JC, 02130JF, 02130JW, |
|  | 02130K3, 02130K8, 02130K9, 02130KC, 02130KF, 02130KW, 02130Z3, 02130Z8, 02130Z9, 02130ZC, |
|  | 02130ZF, 0213444, 0213483, 0213488, 0213489, 021348C, 021348F, 021348W, 0213493, 0213498, |
|  | 0213499, 021349C, 021349F, 021349W, 02134A3, 02134A8, 02134A9, 02134AC, 02134AF, 02134AW, |
|  | 02134D4, 02134J3, 02134J8, 02134J9, 02134JC, 02134JF, 02134JW, 02134K3, 02134K8, 02134K9, |
|  | 02134KC, 02134KF, 02134KW, 02134Z3, 02134Z8, 02134Z9, 02134ZC, 02134ZF |
| Hemodialysis | 5A1D00Z, 5A1D60Z, 5A1D70Z, 5A1D80Z, 5A1D90Z |
| Invasive mechanical ventilation | 5A1935Z, 5A1945Z, 5A1955Z |
| ECMO | 5A0920Z, 5A15223, 5A1522F, 5A1522G, 5A1522H |
| Intra-aortic balloon pump | 5A02110, 5A02210 |

| **Supplementary Table S2. *International Classification of Diseases, Tenth Revisions, Clinical Modification* (ICD-10-CM) codes** | |
| --- | --- |
| **Diagnosis** | **ICD-10-CM codes** |
| Acute kidney injury | N170, N171, N172, N178, N179 |
| Anemia | D460, D461, D4620, D4621, D4622, D464, D500, D508, D509, D510, |
|  | D511, D513, D518, D519, D520, D521, D528, D529, D530, D531, |
|  | D532, D538, D539, D550, D551, D552, D5521, D5529, D553, D558, |
|  | D559, D588, D589, D590, D591, D5910, D5911, D5912, D5913, D5919, |
|  | D592, D594, D598, D599, D6109, D611, D612, D613, D6189, D619, |
|  | D62, D630, D631, D638, D640, D641, D642, D643, D644, D6481, |
|  | D6489, D649 |
| Atrial Fibrillation | I480, I481, I4811, I4819, I482, I4820, I4821, I4828, I4891 |
| Cardiogenic shock | R570, T8111XA, T8111XD, T8111XS |
| Chronic obstructive pulmonary disease | J440, J441, J449 |
| Diastolic heart failure | I5020, I5021, I5022, I5023 |
| End stage renal disease | N186 |
| Heart block | I440, I441, I442, I4430, I4439 |
| Hypothyroidism | E032, E033, E038, E039, E890 |
| Liver disease | K709, K710, K711, K712, K713, K714, K7150, K7151, K716, K717, |
|  | K718, K719, K7589, K759, K769 |
| Perivascular disease | I7389, I739 |
| Prior coronary artery bypass grafts | Z951 |
| Prior percutaneous coronary intervention | Z955 |
| Pulmonary embolism | I2601, I2602, I2609, I2690, I2692, I2693, I2694, I2699, I2782, Z86711 |
| Sleep apnea | G4733 |
| Smoking | F17200, F17208, F17209, F17210, F17218, F17219, Z87891 |
| Stroke | I6000, I6001, I6002, I6010, I6011, I6012, I602, I6020, I6021, I6022, |
|  | I6030, I6031, I6032, I604, I6050, I6051, I6052, I606, I607, I608, |
|  | I609, I610, I611, I612, I613, I614, I615, I616, I618, I619, |
|  | I6200, I6201, I6202, I6203, I621, I629, I63341, I63342, I63343, I63349, |
|  | I6339, I6340, I63411, I63412, I63413, I63419, I63421, I63422, I63423, I63429, |
|  | I63431, I63432, I63433, I63439, I63441, I63442, I63443, I63449, I6349, I6350, |
|  | I63511, I63512, I63513, I63519, I63521, I63522, I63523, I63529, I63531, I63532, |
|  | I63533, I63539, I63541, I63542, I63543, I63549, I6359, I636, I638, I6381, |
|  | I6389, I639, I97810, I97811, I97820, I97821, R29700, R29701, R29702, R29703, |
|  | R29704, R29705, R29706, R29707, R29708, R29709, R29710, R29711, R29712, R29713, |
|  | R29714, R29715, R29716, R29717, R29718, R29719, R29720, R29721, R29722, R29723, |
|  | R29724, R29725, R29726, R29727, R29728, R29729, R29730, R29731, R29732, R29733, |
|  | R29734, R29735, R29736, R29737, R29738, R29739, R29740, R29741, R29742 |
| Systolic heart failure | I5030, I5031, I5032, I5033 |

| **Supplementary Table S3. Characteristics of hospitalizations with coronary artery bypass grafts after overlap weighting** | | |
| --- | --- | --- |
| **Variables** | **Non COVID-19^a^** | **COVID-19** |
| **Age, years** (%) |  |  |
| 18-44 | 5.2 | 5.2 |
| 45-64 | 37.6 | 37.6 |
| ≥ 65 | 57.2 | 57.2 |
| **Sex** (%) |  |  |
| Female | 22.7 | 22.7 |
| Male | 77.3 | 77.3 |
| **Race and ethnicity** (%) |  |  |
| White | 52.9 | 52.9 |
| Hispanic | 28.9 | 28.9 |
| Black | 9.1 | 9.1 |
| Other Race | 9.0 | 9.0 |
| **Type of insurance** (%) |  |  |
| Private | 52.4 | 52.4 |
| Medicare | 32.1 | 32.1 |
| Medicaid | 2.7 | 2.7 |
| Uninsured | 10.4 | 10.4 |
| Other insurance | 2.3 | 2.3 |
| **Deyo comorbidity index** (mean) | 2.00 | 2.00 |
| **Number of organ dysfunctions** (mean) | 1.61 | 1.61 |
| **Comorbid conditions** (%) |  |  |
| Chronic obstructive pulmonary disease | 11.8 | 11.8 |
| Atrial fibrillation | 31.1 | 31.1 |
| End stage renal disease | 8.3 | 8.3 |
| Cardiogenic shock | 19.3 | 19.3 |
| Stroke | 3.7 | 3.7 |
| Pulmonary embolism | 2.1 | 2.1 |
| Systolic heart failure | 21.6 | 21.6 |
| Diastolic heart failure | 13.1 | 13.1 |
| Peripheral vascular disease | 2.9 | 2.9 |
| Sleep apnea | 10.7 | 10.7 |
| Heart block | 4.6 | 4.6 |
| Acute kidney injury | 36.8 | 36.8 |
| **Medical procedures** (%) |  |  |
| Mechanical ventilation | 12.9 | 12.9 |
| Hemodialysis | 6.6 | 6.6 |
| **Year of hospitalization** (mean) | 2021.78 | 2021.78 |
| ^a^ COVID-19: Coronavirus disease 2019 | | |

**R Code used in analysis**

Setup

# Packages
library(tidyverse)
library(moderndive)
library(magrittr)
library(knitr)
library(matlib)
library(readxl)
library(readr)
library(writexl)
library(icdpicr)
library(PSweight)
library(beepr)
library(berryFunctions)
library(lme4)
library(tilting)


# Options
knitr::opts_chunk$set(
 eval = FALSE,
 echo = TRUE,
 error = FALSE,
 fig_align = 'center',
 fig_height = 8,
 fig_width = 8,
 message = FALSE,
 warning = FALSE,
 comment = NULL,
 include = TRUE,
 prompt = FALSE)

# Directory
setwd('~/tabowei')

# Clear

remove(list = ls())

Import

# Directory
setwd("~/tabowei")

# Read
M <- read_rds('cohort_cabg.rds')

PS formula

# propensity score formula
formula_ps <-
 # exposure
 covid ~
 # covariates
 age +
 sex +
 race_ethnicity +
 insurance +
 deyoind +
 oftot +
 copd +
 afib +
 esrd +
 cask +
 stroke +
 pueb +
 shf +
 dhf +
 pvd +
 procimvall +
 prochd +
 sleep_apnea +
 heart_block +
 aki +

year +
 (1 | thcic_id)

Select covariates

# Select
M %<>%
 dplyr::select(
 c(
 # response
 m_h,
 hospital_mortality,
 length_of_stay,
 # exposure
 covid,
 # covariates
 age,
 sex,
 race_ethnicity,
 insurance,
 deyoind,
 oftot,
 copd,
 afib,
 esrd,
 cask,
 stroke,
 pueb,
 shf,
 dhf,
 pvd,
 procimvall,
 prochd,
 sleep_apnea,
 heart_block,
 aki,

year,
 thcic_id))

Mutate

# Drop missing values
M %<>% drop_na

# Convert response to numeric
M %<>% mutate(m_h = as.numeric(m_h))
M %<>% mutate(hospital_mortality = as.numeric(hospital_mortality))
M %<>% mutate(length_of_stay = as.numeric(length_of_stay))

Apply PS weight

# Define group
group <- 'all'

# Define response
response <- c('m_h', 'hospital_mortality', 'length_of_stay')[1]

# Define weight
weights <- c('overlap', 'treated', 'IPW')[1]

# Define y
M %<>% mutate(y = M[[response]])

# PSweight
Qa <-
 PSweight_cl(
 ps.formula = formula_ps,
 trtgrp = NULL,
 yname = response,
 data = M,
 weight = weights,
 delta = 0,
 augmentation = FALSE,
 bootstrap = FALSE,
 bs_level = NULL,
 R = 50,
 out.formula = NULL,
 family = 'binomial',
 nAGQ = 0)

# Summary RR
rr <- summary(Qa, type = 'RR', CI = TRUE)

# Extract RR
rr_e <- exp(rr[['estimates']][1,1])
rr_l <- exp(rr[['estimates']][1,4])
rr_u <- exp(rr[['estimates']][1,5])
rr_p <- rr[['estimates']][1,6]

# Round RR
rr_e <- round(rr_e, digits = 4)
rr_l <- round(rr_l, digits = 4)
rr_u <- round(rr_u, digits = 4)
rr_p <- round(rr_p, digits = 4)

# Format RR
rr_l <- format(rr_l, nsmall = 4, scientific = FALSE)
rr_u <- format(rr_u, nsmall = 4, scientific = FALSE)
rr_p <- format(rr_p, nsmall = 4, scientific = FALSE)
rr_e <- format(rr_e, nsmall = 4, scientific = FALSE)

# Summary RD
rd <- summary(Qa, type = 'DIF', CI = TRUE)

# Extract RD
rd_e <- rd[['estimates']][1,1]
rd_l <- rd[['estimates']][1,4]
rd_u <- rd[['estimates']][1,5]
rd_p <- rd[['estimates']][1,6]

# Round RD
rd_e <- round(rd_e, digits = 4)
rd_l <- round(rd_l, digits = 4)
rd_u <- round(rd_u, digits = 4)
rd_p <- round(rd_p, digits = 4)

# Format RD
rd_e <- format(rd_e, nsmall = 4, scientific = FALSE)
rd_l <- format(rd_l, nsmall = 4, scientific = FALSE)
rd_u <- format(rd_u, nsmall = 4, scientific = FALSE)
rd_p <- format(rd_p, nsmall = 4, scientific = FALSE)

# Labels
label_rr <- paste0(rr_e, ' (', rr_l, ' to ', rr_u, ')')
label_rd <- paste0(rd_e, ' (', rd_l, ' to ', rd_u, ')')

# Tibble
S <-
 tibble(
 weights = weights,
 group = group,
 response = response,
 aRR = label_rr,
 aRD = label_rd)

# Save
write_xlsx(
 x = S,
 path = 'table_three.xlsx',
 col_names = TRUE,
 format_headers = TRUE,
 use_zip64 = TRUE)
